# Supplementary material for: Deep reaction network exploration at a heterogeneous catalytic interface
Source: Nat Commun. 2022 Aug 18;13:4860. doi: 10.1038/s41467-022-32514-7 (PMC9388529; doi:10.1038/s41467-022-32514-7)
Supplement: Supplementary file 1 — Supplementary Information [file 41467_2022_32514_MOESM1_ESM.pdf]

Deep Reaction Network Exploration at a Heterogeneous Catalytic Interface

Supporting Information

Qiyuan Zhao[†], Yinan Xu[†], Jeffrey Greeley,[*] and Brett M. Savoie[*]

Davidson School of Chemical Engineering, Purdue University, West Lafayette, IN, 47906

E-mail: jgreeley@purdue.edu; bsavoie@purdue.edu

1 Comparisons between different levels of DFT theory in computing energies of the Ga^{3+}-containing species

To evaluate the potential errors associated with the choice of DFT level, a comparison was made between B3LYP/6-31G, B3LYP-D3/6-311G**, B3LYP-D3/TZVP, and ωB97XD/TZVP† (TZVP† refers to a combination of def2-TZVP used for gallium and TZVP used for other atoms) for three types of elementary reactions. The range-separated functional ωB97XD/TZVP† was adopted as an accurate baseline.[?] The error associated with performing high-level single-points on low-level geometries was investigated by including the results for ωB97XD/TZVP† calculations on geometries optimized at the B3LYP/6-31G level of theory (ωB97XD/TZVP†// B3LYP/6-31G curve in Fig. S1).

Compared to the ωB97XD/TZVP† baseline energies, large deviations are observed for the reaction energies computed at the B3LYP/6-31G level, including both B3LYP/6-31G-only calculations and the hybrid ωB97XD/TZVP†//B3LYP/6-31G calculations. A dramatic im-

provement in accuracy is observed by adding the D3 dispersion correction and increasing the size of the basis set. B3LYP-D3/TZVP and B3LYP-D3/6-311G** have similar performance on the calculations of activation energy and reaction energy. Due to the lower computational cost and comparable accuracy, the B3LYP-D3/6-311G** level of theory was used to refine the geometries and recalculate the energies for the reactions reported in the main text.

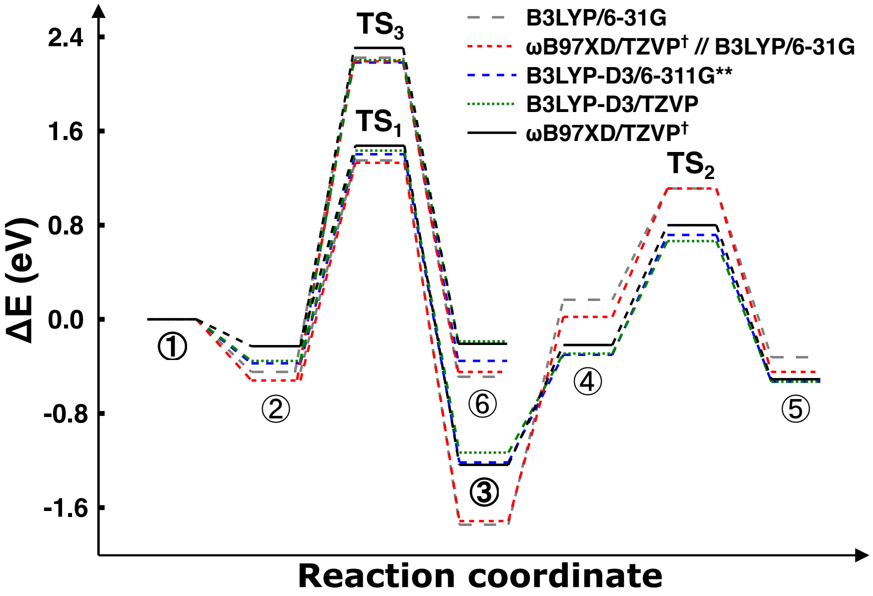

Figure S1: Potential energy diagram of type *I-III* reactions computed at different levels of theory. TZVP† refers to a combination of def2-TZVP on gallium and TZVP on other atoms. Sites refer to: 1. Ga-ethyl, 2. Ga-ethyl and ethene, 3. Ga-butyl, 4. Ga-butyl and ethene, 5. Ga-ethyl and 1-butene, 6. Ga-vinyl and ethane.

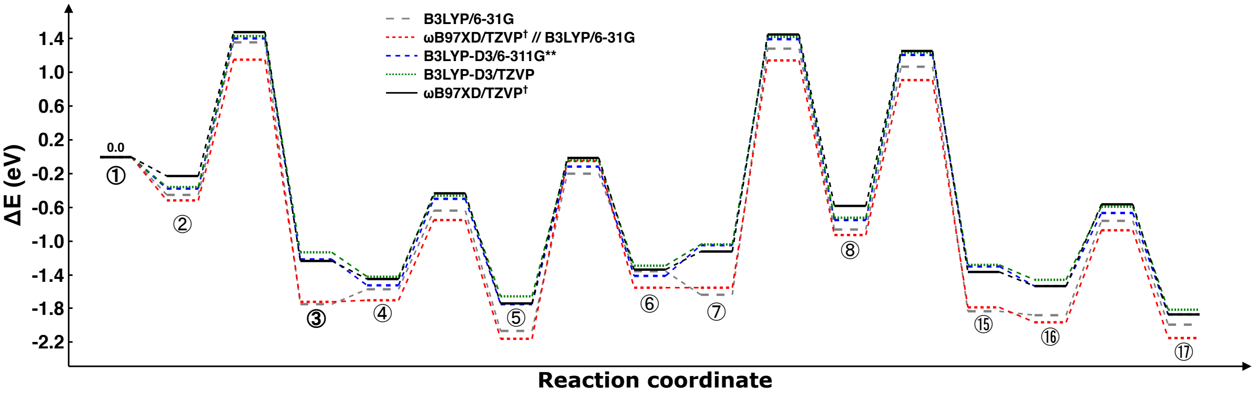

Figure S2: Potential energy diagram of isobutene formation pathway reported in main text Figure 4 computed at five different levels of theory.

Similar comparisons were also made on the isobutene formation pathway (Fig. S2). The

same prediction trends for different level of theory are observed which further supports the use of B3LYP-D3/6-311G** in the cluster model to refine the geometries and energies. Due to the geometric differences of the cluster and periodic models, refining the functional and basis set will only increase the accuracy up to a point. Refining the reaction network afterward using a periodic model seems like a workable near-term solution when surface effects are expected to be comparable to the accuracy of DFT. Alternatively, there are few barriers to extending reaction exploration algorithms like YARP to be compatible with periodic codes.

2 Benzene formation pathways

In the main text, acetylene is treated as a coke precursor due to its facility for further aromatization and dehydrogenation.[1–3] Using the cluster model and acetylene as the reactant, we have characterized the transition states associated with a three-step mechanism for Ga-catalyzed formation of benzene from acetylene (Fig. S3a). This pathway was discovered during a non-comprehensive exploration of benzene production pathways using YARP and is only provided as an illustration of a possible Ga-mediated pathway. In this pathway, two type I reactions between acetylene and Ga-vinyl result in a longer C6 species (with $\Delta G^{\dagger}$ of 41.83 and 34.82 kcal/mol, respectively), which is followed by the liberation of benzene and H_2 elimination through a nearby hydroxyl group ($\Delta G^{\dagger} = 19.19$ kcal/mol). For comparison, non-catalytic acetylene cyclization forming benzene requires an activation energy of 49.77 kcal/mol when calculated at the same level of theory (Fig. S3b).

3 Hydrogen gas formation pathway

Following the formation of Ga-hydride, H_2 gas can be produced via protons from nearby hydroxyl groups (Fig. S3c). The activation barriers of Ga-hydride formation through β-hydride elimination, and H_2 generation steps are 56.43 and 37.69 kcal/mol, respectively. However, the competing β-hydride transfer forming Ga-ethyl is much more favorable involving an ac-

tivation barrier of 34.43 kcal/mol. Therefore, the formation of H_2 gas is considered unlikely. This is also consistent with previous experimental results.[4]

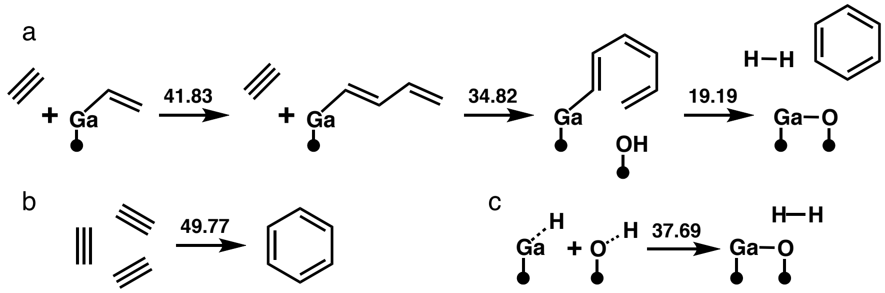

Figure S3: Additional pathways for bezene and hydrogen gas production. Reaction pathways of (a) Single-site Ga catalyzed benzene formation, (b) Gas-phase benzene formation and (c) H_2 formation by closing a Ga site. Single point energies are computed at B3LYP-D3/6-311G** level of theory and the unit is kcal/mol.

4 Extending the cluster model results to Al^{3+} single site

To demonstrate the predictive capability of network exploration using the YARP-cluster approach, the network for ethylene oligomerization was redone with Ga^{3+} substituted with Al^{3+}. The Al^{3+} site exhibits overall very similar for ethylene insertion, β-hydride transfer, and α-hydride transfer, suggesting that silica-supported Al^{3+} is also a viable catalyst for ethylene oligomerization (Fig. S4).

5 Additional details of YARP exploration results

Table S1: Free energies of activation for each edge (reaction) in Figure 2 of the main text computed at B3LYP/6-31G level.

Reactant node	Product node	$\Delta G^\dagger$ (kcal/mol)
0	1	59.84

0	2	76.07
0	4	24.43
0	5	44.08
0	6	35.37
0	7	28.95
0	8	58.66
0	9	56.38
0	12	44.80
0	14	46.34
0	18	56.43
0	20	44.09
0	21	47.95
0	22	52.98
0	23	30.77
0	24	40.74
0	25	45.68
0	26	29.88
1	0	51.35
1	5	54.52
1	6	64.82
1	7	53.16
1	15	44.65
1	17	69.21
1	22	65.35
1	23	58.72
1	26	56.21

1	27	41.30
1	29	67.84
3	1	61.59
3	4	51.81
3	15	66.46
3	16	44.79
3	17	60.12
3	18	58.16
4	0	45.20
4	1	76.56
4	19	61.11
5	0	36.05
5	1	76.44
5	10	61.31
6	0	35.13
6	1	75.07
6	2	71.18
6	3	65.18
6	13	62.06
16	0	34.43
23	1	65.99
23	28	65.99
26	4	40.75
26	15	60.85

Table S2: Single point energies of each site and transition state in Figure 5 of the main text. The cluster and periodic results were calculated at the B3LYP-D3/6-311G(d,p) and BEEF-VdW/PAW levels of theory, respectively. Ref refers to energies with respect to the single-point energy of Ga-ethyl plus a gaseous ethylene molecule.

Sites	Cluster (Hartree)	Cluster (with ref., eV)	Periodic (with ref., eV)
1	-5466.849	0.000	0.00
2	-5545.478	-0.370	-0.47
3	-5545.508	-1.205	-1.03
4	-5624.134	-1.505	-1.44
5	-5624.143	-1.732	-1.36
6	-5624.130	-1.397	-1.30
7	-5545.502	-1.033	-0.98
8	-5545.491	-0.744	-0.83
9	-5427.526	-0.238	-0.25
10	-5506.154	-0.592	-0.65
11	-5506.179	-1.282	-1.11
12	-5584.806	-1.607	-1.44
13	-5584.817	-1.902	-1.62
14	-5624.146	-1.764	
15	-5545.511	-1.284	
16	-5624.135	-1.522	
17	-5624.147	-1.847	
3a	-5545.481	-0.152	
4*	-5544.238	-0.470	
5*	-5544.213	0.447	
3b	-5624.133	-1.208	
3c	-5624.133	-1.208	
3d	-5506.149	-0.361	
2–>3	-5545.412	1.395	1.11
4–>5	-5624.097	-0.488	-0.52
5–>6	-5624.083	-0.110	-0.47
7–>8	-5545.413	1.380	1.09
10–>11	-5506.077	1.497	1.03
12–>13	-5584.762	-0.408	-0.40
6–>14	-5624.094	-0.110	
8–>15	-5545.419	1.202	
16–>17	-5624.103	-0.662	
2–>3a	-5545.384	2.168	
4–>3b	-5624.038	1.091	
6–>3c	-5624.037	1.117	
10–>3d	-5506.058	1.987	
4*–>5*	-5544.176	1.205	

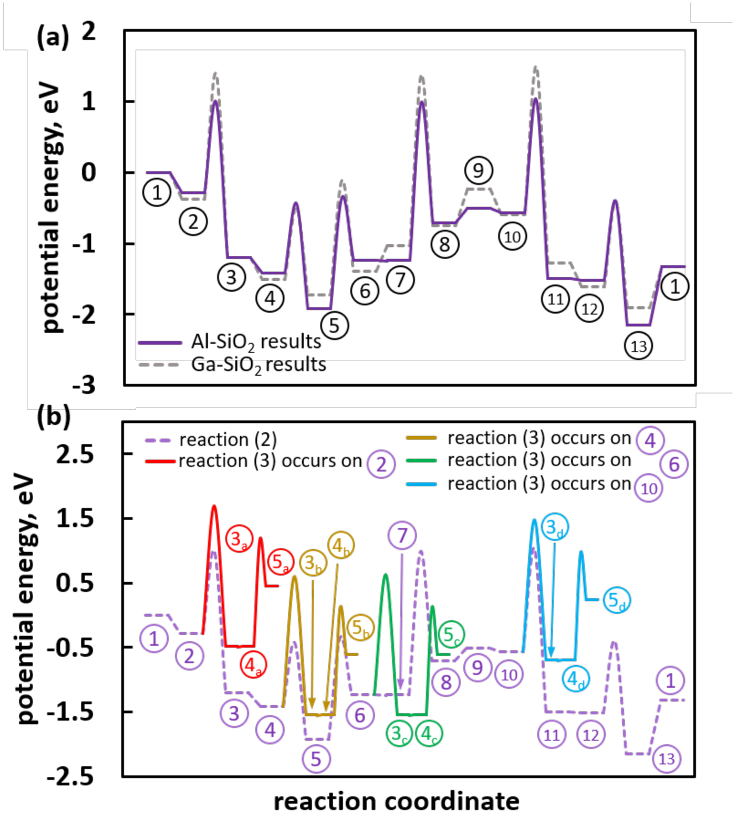

Figure S4: Alkane oligomerization on silica supported Al^{3+} sites. Energy profiles of (a) ethylene oligomerization, isomerization, cracking, and (b) the formation of light alkanes on the Al^{3+} single site using the silica cluster model. The same species numbers and structures as in the main text Figure 4 are used, with the exception that the metal site identity of Ga is replaced by Al.

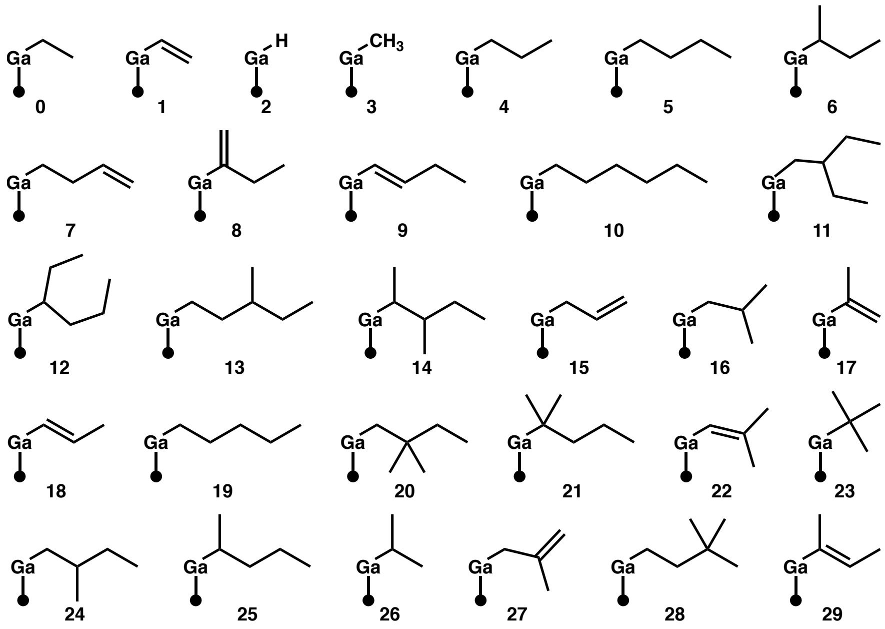

Figure S5: All products in the reaction network downstream of Ga-ethyl formation predicted by YARP. Labels correspond to those in Figure 2 and to the in-text references within the main text.

6 Ga^{3+} single site on periodic silica model

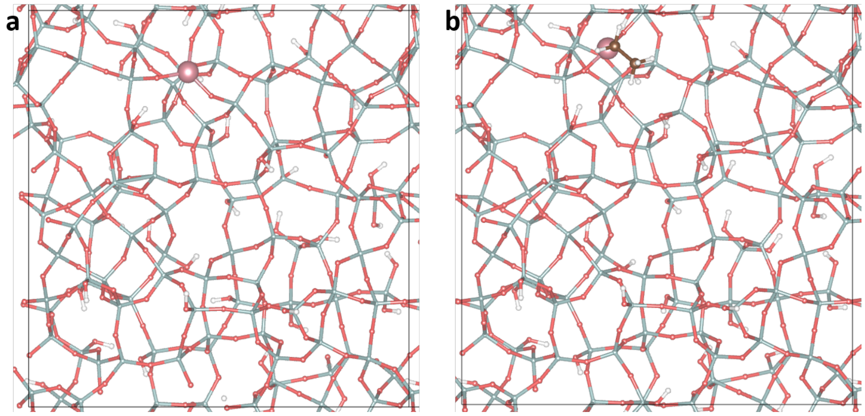

Figure S6: Snapshots of the amorphous silica model. Top view with (a) the empty Ga single site highlighted and (b) the Ga-ethyl intermediate.

The periodic Ga^{3+}-silica model used here was adopted from previous work on ethylene oligomerization.[5] The silica model, originally developed by Comas-Vives, has a large unit cell (21.6 × 21.6 × 14.4 Å, with a vacuum thickness of 20 Å) containing 402 atoms, and the amorphous morphology originates from an annealing process using classical molecular dynamics and direct condensation of the adjacent silanol pairs. As analyzed by Comas-Vives, the amorphous characteristics are confirmed by the high variability of the condensation energy and the Si-O bond lengths of the resulting siloxane rings, which range from 1 – 322 kJ /mol and 1.65 – 1.75 Å, respectively.[6] Our previous work compared the reactivity of two types of Ga single sites: a stretched three-coordinated and a constrained four-coordinated Ga sites. The four-coordinated one consistently exhibits higher barriers of ethylene insertion and β-hydride transfer steps due to strong steric hindrance effects. Therefore, the stretched three-coordinated site is used in this study for the analysis of other elementary reactions.[5] This work focuses on the Ga-ethyl moiety, an intermediate of the activation process of empty three-coordinated Ga site, which initiates the Cossee-Arlman oligomerization cycle.[5] Figure S6 shows the unit cell of the periodic silica model with the highlighted location of the Ga site.

7 Schematics of reaction intermediates and transition states on periodic model

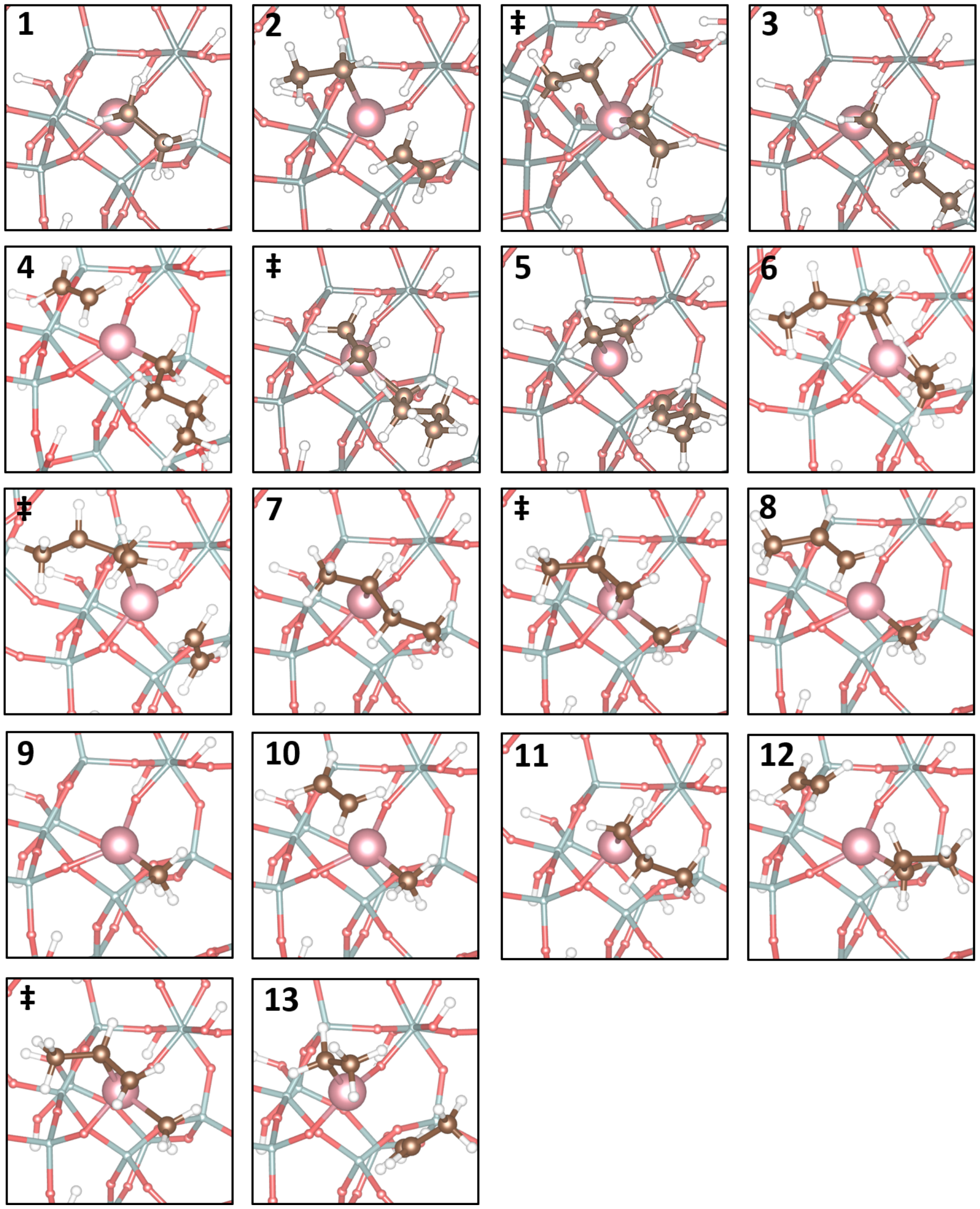

Figure S7: Schematics of reaction intermediates and transition states on the periodic model. The Ga atom is highlighted, brown=carbon, white=hydrogen, red=oxyen, and green=silicon.

Supplementary References

(1) Kopinke, F. D.; Zimmermann, G.; Reyniers, G. C.; Froment, G. F. Relative rates of coke formation from hydrocarbons in steam cracking of naphtha. 2. paraffins, naphthenes, mono-, di-, and cycloolefins, and acetylenes. *Ind. Eng. Chem. Res.* **1993**, *32*, 56–61.

(2) Cai, H.; Krzywicki, A.; Oballa, M. C. Coke formation in steam crackers for ethylene production. *Chem. Eng. Process.* **2002**, *41*, 199–214.

(3) Wauters, S.; Marin, G. Kinetic modeling of coke formation during steam cracking. *Ind. Eng. Chem. Res.* **2002**, *41*, 2379–2391.

(4) LiBretto, N. J.; Xu, Y.; Quigley, A.; Edwards, E.; Nargund, R.; Vega-Vila, J. C.; Caulkins, R.; Saxena, A.; Gounder, R.; Greeley, J.; Zhang, G.; Miller, J. T. Olefin oligomerization by main group Ga^{3+} and Zn^{2+} single site catalysts on SiO2. *Nat. Commun.* **2021**, *12*.

(5) Xu, Y.; LiBretto, N.; Zhang, G.; Miller, J.; Greeley, J. First Principles Analysis of Ethylene Oligomerization on Single-site Ga^{3+} Catalysts Supported on Amorphous Silica. *ACS Catalysis* **2022**, *12*, 5416–5424.

(6) Comas-Vives, A. Amorphous SiO_2 surface models: energetics of the dehydroxylation process, strain, ab initio atomistic thermodynamics and IR spectroscopic signatures. *Phys. Chem. Chem. Phys.* **2016**, *18*, 7475–7482.
